# TGF‐β/Smad and JAK/STAT pathways are involved in the anti‐fibrotic effects of propylene glycol alginate sodium sulphate on hepatic fibrosis

**DOI:** 10.1111/jcmm.15175

**Published:** 2020-03-31

**Authors:** Shizan Xu, Yuqing Mao, Jianye Wu, Jiao Feng, Jingjing Li, Liwei Wu, Qiang Yu, Yuting Zhou, Jie Zhang, Jiaojiao Chen, Jie Ji, Kan Chen, Fan Wang, Weiqi Dai, Xiaoming Fan, Chuanyong Guo

**Affiliations:** ^1^ Department of Gastroenterology Putuo People's Hospital Tongji University School of Medicine Shanghai China; ^2^ Department of Gastroenterology Jinshan Hospital of Fudan University Shanghai China; ^3^ Department of Gastroenterology Shanghai Tenth People’s Hospital Tongji University School of Medicine Shanghai China; ^4^ Shanghai Tenth Hospital School of Clinical Medicine of Nanjing Medical University Shanghai China; ^5^ Department of Gerontology Shanghai General Hospital Shanghai Jiao Tong University School of Medicine Shanghai China; ^6^ Department of Oncology Shanghai General Hospital Shanghai Jiao Tong University School of Medicine Shanghai China; ^7^ Department of Gastroenterology Zhongshan Hospital of Fudan University Shanghai China; ^8^ Shanghai Institute of Liver Diseases Zhongshan Hospital of Fudan University Shanghai China; ^9^ Shanghai Tongren Hospital Shanghai Jiao Tong University School of Medicine Shanghai China

**Keywords:** autophagy, JAK2/STAT3, liver fibrosis, propylene glycol alginate sodium sulphate, TGF‐β1/Smad2/3

## Abstract

Liver fibrosis, a consequence of unhealthy modern lifestyles, has a growing impact on human health, particularly in developed countries. Here, we have explored the anti‐fibrotic effects of propylene glycol alginate sodium sulphate (PSS), a natural extract from brown algae, in fibrotic mice and cell models. Thus, we established bile duct ligature and carbon tetrachloride mouse models and LX‐2 cell models with or without PSS treatment. Liver pathological sections and the relevant indicators in serum and liver tissues were examined. PSS prevented hepatic injury and fibrosis to a significant extent, and induced up‐regulation of matrix metalloproteinase‐2 and down‐regulation of tissue inhibitor of metalloproteinase‐1 through suppressing the transforming growth factor β1 (TGF‐β1)/Smad pathway. PSS additionally exerted an anti‐autophagy effect through suppressing the Janus kinase (JAK) 2/transducer and activator of transcription 3 (STAT3) pathway. In conclusion, PSS prevents hepatic fibrosis by suppressing inflammation, promoting extracellular matrix (ECM) decomposition and inactivating hepatic stellate cells through mechanisms involving the TGF‐β1/Smad2/3 and JAK2/STAT3 pathways in vivo and in vitro.

## 1 INTRODUCTION

Liver fibrosis or scarring, a damage‐induced reaction to heal wounds by encapsulating the injury, is a significant cause of morbidity worldwide and contributes to 45% mortality in developed countries.^1^, ^2^ Following chronic injury, the extracellular matrix (ECM) accumulates, mainly due to activation of hepatic stellate cells (HSC).^3^, ^4^ HSCs, normally quiescent cells that store vitamin A, can be proliferated in response to liver injury. This process goes along with release of the transforming growth factor β1 (TGF‐β1) from activated Kupffer cells and is characterized by the appearance of smooth muscle α‐actin (α‐SMA).^1^, ^3^, ^5^, ^6^ Collagen type I (Col‐1), a crucial constituent of the fibril‐forming matrix, increases gradually in a background of HSC activation and presents a relatively late event in hepatic fibrosis.^1^, ^7^ Matrix metalloproteinase‐2 (MMP‐2), a basement membrane protease and type IV collagenase, degrades the extracellular matrix and acts against fibrosis. Conversely, binding of tissue inhibitors of metalloproteinase‐1 (TIMP‐1) to interstitial collagenases suppresses degradation of the accumulating matrix and prevents clearance of HSCs.^8^ Smad2 and Smad3 proteins are phosphorylated with release of TGF‐β1 and, in turn, promoting its activity and HSC activation.^1^

Propylene glycol alginate sodium sulphate (PSS) is a heparinoid drug initially identified in brown algae by a Chinese scientist.^9^, ^10^ Studies have reported protective effects of PSS on concanavalin A and ischaemia reperfusion‐induced liver injury models that are attributed to its anti‐inflammatory ability and activity in reducing blood viscosity.^11^, ^12^ However, no literature has studied the efficiency of PSS in liver fibrosis. Interestingly, low–molecular‐weight heparin is reported to exert anti‐fibrotic effects,^13^ bringing forward the hypothesis that PSS could prevent hepatic fibrosis in mice.

Autophagy is a well‐known dynamic cellular process that plays an important role in fibrosis.^14^ Activation of HSCs has been shown to facilitate autophagic flux,^15^, ^16^ and involvement of specific molecular pathways, including Janus kinase (JAK) 2/signal transducer and activator of transcription 3 (STAT3), was in regulation of autophagy as reported.^17^, ^18^ Furthermore, several studies have investigated the roles of STAT3 in fibrosis, both in vitro and in vivo.^19^, ^20^ Based on the findings to date, JAK2/STAT3 pathway has been highlighted as a potential anti‐fibrosis target in clinical therapy.

Bile duct ligature (BDL) and carbon tetrachloride (CCl_4_) mice models and activated LX‐2 cells models are widely established for the assessment of liver fibrosis and cirrhosis.^4^, ^21^ As few effective treatments exist for hepatic cirrhosis, end stage of fibrosis or precancerous stages of hepatocellular carcinoma, more research focus is required on earlier fibrosis.^22^ In this study, the above fibrotic models were employed to investigate the effects of PSS following chronic administration and the underlying mechanisms.

## 2 MATERIALS AND METHODS

### 2.1 Drugs and reagents

Propylene glycol alginate sodium sulphate was purchased from Dalian Tianyu Pharmaceuticals Co., Ltd and disposed in saline to obtain different drug doses of 12.5, 25 and 50 mg/kg, and stored at 4°C.^12^ CCl_4_ was acquired from Sinopharm. Microplate test kits of alanine aminotransferase (ALT) and aspartate aminotransferase (AST) were obtained from Nanjing Jiancheng Bioengineering Institute (Jiancheng Biotech). Antibodies against IL‐6, Col‐1, α‐SMA, TGF‐β1, MMP‐2, TIMP‐1, Beclin‐1, p62, Smad2 and Smad3 were purchased from Proteintech, and those against p‐Smad2, p‐Smad3, JAK2, STAT3 and p‐STAT3 were purchased from Cell Signaling Technologies. The polymerase chain reaction (PCR) kit was acquired from Takara Biotechnology.

### 2.2 Animal experiments

All animal experiments were conducted following the institutional guidelines and approved by the Animal Care and Use Committee of Shanghai Tongji University, China. Healthy male C57 mice (20‐22 g) 6 weeks of age, provided by Shanghai Laboratory Animal Co., Ltd, were maintained in tidy cages with free access to food and water, and equilibrated for a week before experimental enrolment.

In the BDL experiment, 48 mice were divided into six groups: (1) normal control (NC), (2) sham, (3) BDL, (4) BDL + PSS (12.5), (5) BDL + PSS (25) and (6) BDL + PSS (50). The BDL operation was performed as described previously.^21^ In groups (4), (5) and (6), mice were intraperitoneally injected with the indicated doses of PSS at 12.5, 25 and 50 mg/kg, respectively, and mice in groups (1), (2) and (3) were treated with the same volume of saline twice a week for two subsequent weeks. In the CCl_4_ experiment, 40 CCl_4_ model mice were correspondingly divided into five groups: (1) vehicle‐treated control, (2) CCl_4_, (3) CCl_4_ + PSS (12.5), (4) CCl_4_ + PSS (25) and (5) CCl_4_ + PSS (50). Mice were treated with CCl_4_ with or without PSS for a total of 12 weeks. In order to induce hepatic fibrosis, CCl_4_ dissolved in olive oil (10%) was injected intraperitoneally (1.0 mL/kg) twice a week. The dose of PSS in groups (3), (4) and (5) was 12.5, 25 and 50 mg/kg, respectively, in CCl_4_ experiment.

Following the experiment, mice were killed by CO_2_ inhalation. Blood samples were collected into 20 µL heparin (1000 U/mL), and serum samples were obtained by centrifugation for 10 minutes at 4500 *g* and 4°C. Liver samples were excised and processed after mice were killed, and stored at −80°C.

### 2.3 Cell culture

Human primary HSC cell line, LX‐2, was purchased from Cell Bank of Type Culture Collection of the Chinese Academy of Sciences and maintained in Dulbecco's modified Eagle Medium (DMEM) containing penicillin, streptomycin and foetal bovine serum as described.^4^ HSC cells have been shown to be a primary effector of hepatic fibrosis, and in our study, LX‐2 cells were treated with 10 ng/mL TGF‐β1 (PeproTech) to be activated after 24 hours.^4^, ^23^

### 2.4 Biochemical measurements and enzyme‐linked immunosorbent assay (ELISA)

ALT and AST activities in serum were determined according to standard protocols using the test kits and an automated chemistry analyser (Olympus AU1000). Serum α‐SMA and Col‐1 were detected with commercial ELISA kits (R&D Systems).

### 2.5 Histological analysis

Liver specimens were washed with saline, followed by fixation in 10% formalin and embedding in paraffin. Specimens 5 µm thick were subjected to haematoxylin and eosin (HE), Sirius Red and Mason's trichrome staining to evaluate hepatic fibrosis under a light microscope. The degree of liver fibrosis was examined by a specialist blinded to sample information according to strict criteria. Fibrosis scores and related stages were as follows: 0, no fibrosis; 1, perisinusoidal or periportal fibrosis; 2, perisinusoidal and portal/periportal fibrosis; 3, bridging fibrosis; and 4, cirrhosis.^24^

### 2.6 Immunohistochemistry

Immunostaining of liver sections embedded in paraffin was performed with primary antibodies against TGF‐β1, Col‐1, α‐SMA, MMP‐2, TIMP‐1, Beclin‐1, p62, p‐smad2, p‐smad3 and p‐STAT3 as reported.^25^ Sections were subsequently incubated with the corresponding secondary antibodies. Bound antibodies were observed under a light microscope and images obtained with a digital camera (Leica Wetzlar) after incubation with a peroxidase substrate (DAB) kit (Vector).

### 2.7 Western blot analysis

Protein samples were obtained using standard protocols and the concentrations determined with a bicinchoninic acid protein assay kit (Solarbio). Western blot analysis of IL‐6, TGF‐β1, Col‐1, α‐SMA, MMP‐2, TIMP‐1, Beclin‐1, p62, p‐smad2, p‐smad3, p‐STAT3 and β‐actin (control) was conducted with standard methods. Different band quantities were detected via an Odyssey two‐colour infrared laser imaging system (LI‐COR Biosciences).

### 2.8 Reverse transcription (RT)‐PCR

Total RNA was obtained with TRIzol reagent (Takara) using standard protocols. After purity determination, single‐stranded cDNA was synthesized via reverse transcription using a ThermoScript RT‐PCR system (Invitrogen). Related gene expression was examined with a ViiA™ 7 Real‐Time PCR System (Applied Biosystems). All the detections were performed in duplicate, and experiments were repeated at least three times. The primers used are listed in Table 1.

**Table 1:** The primers used in the study

Gene		Primers sequence (5′–3′)
α‐SMA	Forward	CCCAGACATCAGGGAGTAATGG
	Reverse	TCTATCGGATACTTCAGCGTCA
Col Ⅰ	Forward	CAATGGCACGGCTGTGTGCG
	Reverse	AGCACTCGCCCTCCCGTCTT
MMP‐2	Forward	GGACAAGTGGTCCGTGTAAA
	Reverse	CCGACCGTTGAACAGGAAGG
TIMP‐1	Forward	CGAGACCACCTTATACCAGCG
	Reverse	ATGACTGGGGTGTAGGCGTA
TGF‐β1	Forward	CCACCTGCAAGACCATCGAC
	Reverse	CTGGCGAGCCTTAGTTTGGAC
Beclin‐1	Forward	ATGGAGGGGTCTAAGGCGTC
	Reverse	TGGGCTGTGGTAAGTAATGGA
p62	Forward	GAGGCACCCCGAAACATGG
	Reverse	ACTTATAGCGAGTTCCCACCA
β‐Actin	Forward	GGCTGTATTCCCCTCCATCG
	Reverse	CCAGTTGGTAACAATGCCATGT

### 2.9 Detection of cell viability and the TGF‐β/Smad and JAK/STAT pathways in vitro

We detected the viable cells before and after the activation of TGF‐β1 with the CCK8 assay (Dojindo Laboratories). LX‐2 cells were seeded into 96‐well plates for 48 hours, followed with the addition of PSS at concentration of 0, 1.0, 2.5, 5.0, 7.5, 10.0, 12.5 and 15.0 μg/mL for 24 hours. CCK8 assay was used to detect cell viability according to the manufacturer's instructions, assuming the absorbance of the cells with 0 μg/mL PSS as 100%. To further study whether TGF‐β/Smad and JAK/STAT pathways were involved in our study, LX‐2 cells, cultured in 6‐well plates, were divided into three groups in our vitro experiment. LX‐2 cells, cultured in 6‐well plates, were divided into three groups in our vitro experiment: (a) normal group: cells were treated with DMEM only; (b) TGF‐β1–treated group: cells were treated with TGF‐β1 without PSS administration; and (c) PSS group: cells were treated with TGF‐β1 and PSS at the half‐maximum inhibition concentration (IC50) that could be gained in accordance with the above studies.

### 2.10 Immunofluorescence (IF)

LX‐2 cells were cultured, and the IF staining against p‐Smad2 and p‐Stat3 was carried based on a recent study.^27^

### 2.11 Statistical analysis

Results were presented as means ± standard deviation (SD) of at least three repeated independent experiments. Statistical analysis was conducted with the Student *t* test. *P*‐values < .05 were considered significant.

## 3 RESULTS

### 3.1 Protection of liver against the effects of BDL and CCl_4_ by PSS

**Figure 1:**
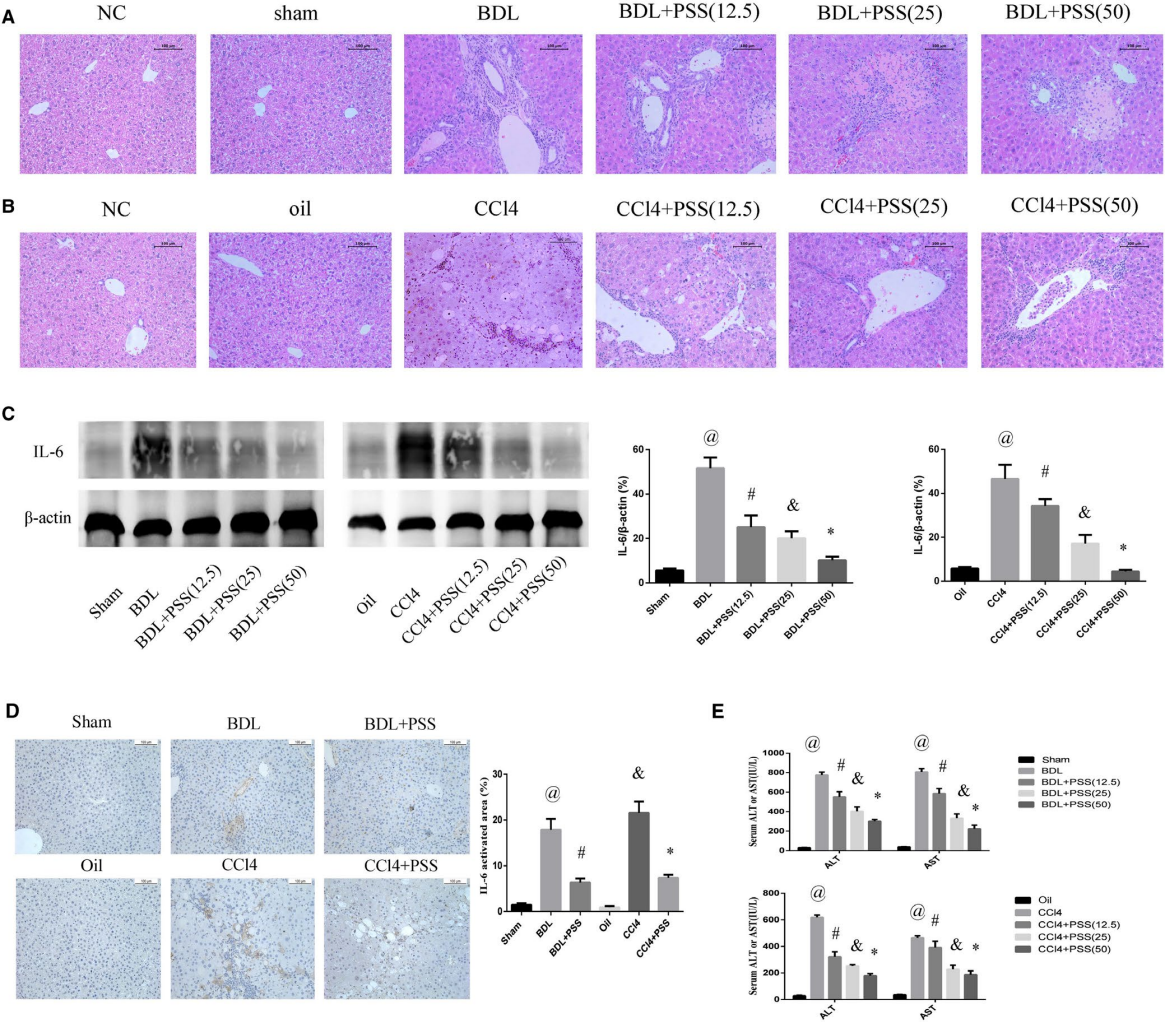
Protective effects of PSS on liver in BDL and CCl4 mouse models. A, HE staining of liver tissues in BDL experiment. Original magnification, 200×. B, HE staining of liver tissues in CCL4 experiment. Original magnification, 200×. C, IL‐6 protein expression was detected via Western blot. D, IHC staining of IL‐6 in liver tissues. We chose the PSS dose of 50 mg/kg as the representative dose in the subsequent experiment. Data were expressed as means ± SD (n = 8). ^@^ *P* < .05 for BDL vs sham group, ^#^ *P* < .05 for BDL + PSS vs BDL group, ^&^ *P* < .05 for CCl4 vs oil group, *P < .05 for CCl4 + PSS vs CCl4 group. E, Suppression of serum ALT and AST levels. Above data are presented as means ± SD (n = 8). In BDL experiment, ^@^ *P* < .05 for BDL vs sham group, ^#^ *P* < .05 for BDL + PSS (12.5) vs BDL group. ^&^ *P* < .05 for BDL + PSS (25) vs BDL group, *P < .05 for BDL + PSS (50) vs BDL group. In CCl4 experiment, ^@^ *P* < .05 for CCl4 vs oil group, ^#^ *P* < .05 for CCl4 + PSS (12.5) vs CCl4 group. ^&^ *P* < .05 for CCl4 + PSS (25) vs CCl4 group, *P < .05 for CCl4 + PSS (50) vs CCl4 group

Sham operation or oil injection led to no significant differences in HE staining when compared with the NC group, and it was consistent with a recent research.^21^ The results of HE staining in the experimental models revealed considerably greater histological changes in the BDL group relative to the sham group. We observed a marked reduction in these changes after administration of different doses of PSS (Figure 1A). Injection of CCl_4_ for 12 weeks caused significant damage to liver, compared with the oil vehicle group, which was markedly prevented with simultaneous PSS treatment (Figure 1B). Next, we examined the expression of IL‐6, an important inflammatory factor in liver injury, via Western blot (Figure 1C) and immunohistochemistry analyses (Figure 1D). Notably, IL‐6 expression increased in the BDL and CCl_4_ groups was inhibited by PSS. To further confirm the protective function of PSS, ALT and AST activities in BDL operation and repeat CCl_4_ injection groups were examined. Notably, the significantly elevated activities of ALT and AST observed in both fibrosis models were reduced in the presence of PSS (Figure 1E).

### 3.2 PSS prevents hepatic fibrosis induced by BDL and CCl_4_

**Figure 2:**
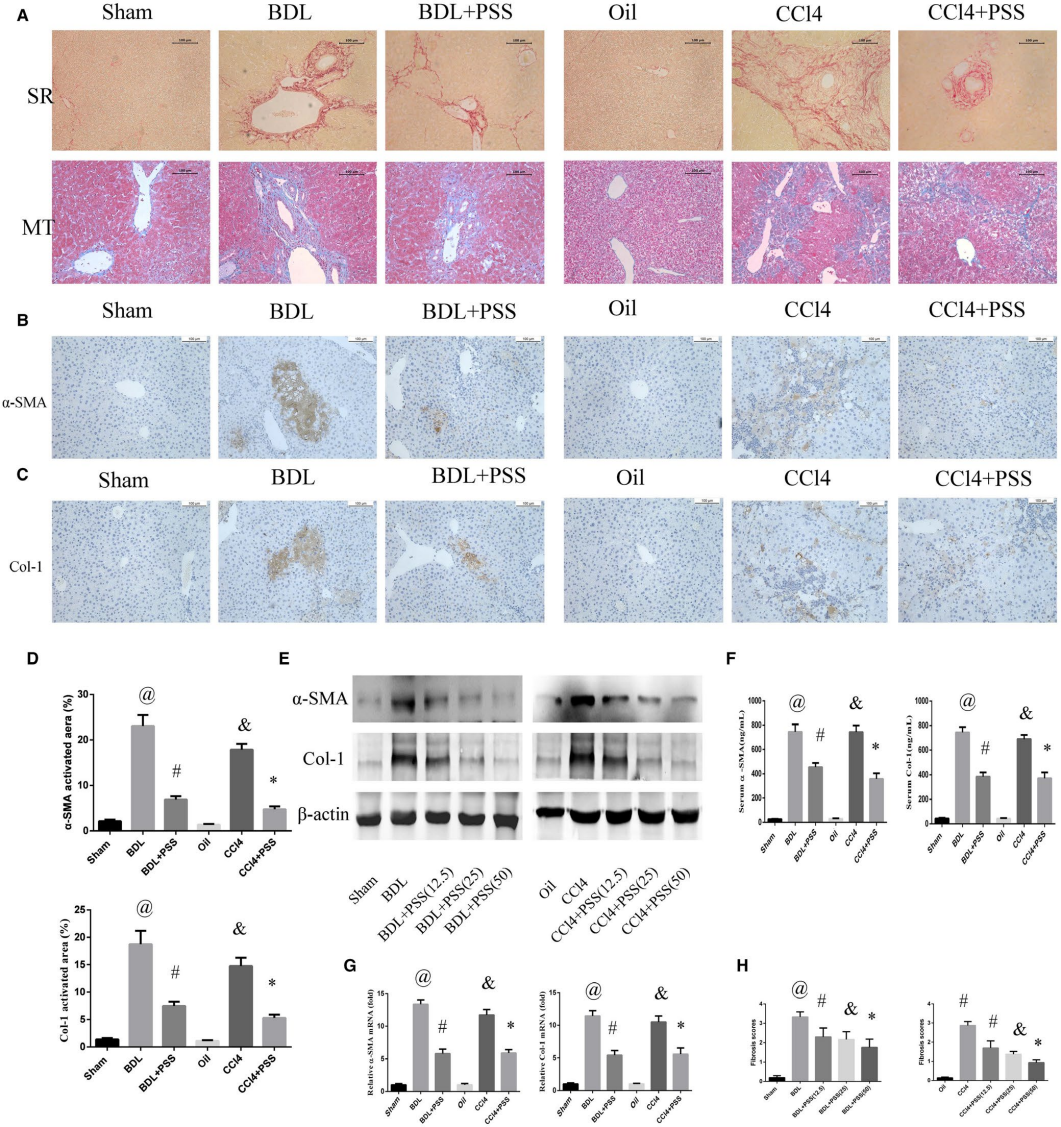
Propylene glycol alginate sodium sulphate prevents hepatic fibrosis induced by BDL and CCl4. A, Sirius red and Masson's trichrome staining in liver tissues. Original magnification, 200×. B, IHC staining of anti‐α‐SMA in liver tissues. C, IHC staining of anti‐Col I in liver tissues. D, Labelling index data for (B) and (C). E, Western blotting of α‐SMA and Col I. F, Serum α‐SMA and Col I levels. G, Expression of α‐SMA and Col I mRNA. Data of (D), (F) and (G) were presented as means ± SD (n = 8), ^@^ *P* < .05 for BDL vs sham group, ^#^ *P* < .05 for BDL + PSS vs BDL group, ^&^ *P* < .05 for CCl4 vs oil group, *P < .05 for CCl4 + PSS vs CCl4 group. H, Fibrosis scores of different groups. Data were shown as means ± SD (n = 8). In BDL experiment, ^@^ *P* < .05 for BDL vs sham group, ^#^ *P* < .05 for BDL + PSS (12.5) vs BDL group. ^&^ *P* < .05 for BDL + PSS (25) vs BDL group, *P < .05 for BDL + PSS (50) vs BDL group. In CCl4 experiment, ^@^ *P* < .05 for CCl4 vs oil group, ^#^ *P* < .05 for CCl4 + PSS (12.5) vs CCl4 group. ^&^ *P* < .05 for CCl4 + PSS (25) vs CCl4 group, *P < .05 for CCl4 + PSS (50) vs CCl4 group

To evaluate liver fibrosis, we examined deposition of Col‐1 with Sirius red staining and collagen fibre with Masson's trichrome staining. Hepatic fibrosis was markedly amplified after the BDL operation. In contrast, PSS‐treated mice displayed pronounced reduction in fibrosis (Figure 2A). In the CCl_4_ model, PSS consistently prevented fibrosis to a significant extent. After BDL operation or CCl_4_ injection, the expression of Col‐1 and α‐SMA was significantly increased. Up‐regulation of Col‐1 and α‐SMA was blocked upon PSS treatment, as observed from immunohistochemical staining data (Figure 2B‐D). Western blot analysis consistently indicated preventive functions of PSS against hepatic fibrosis (Figure 2E). The efficiency of PSS treatment was further validated via ELISA of serum α‐SMA and Col‐1 (Figure 2F) and quantitative PCR (Figure 2G). The serum level of α‐SMA and Col‐1 increased in BDL or CCl4 group, and they were reduced in PSS‐treated groups. The mRNA expression of α‐SMA and Col I showed consistent results.

Fibrosis scores were obtained through rigorous evaluation by a specialist based on histological staining (Figure 2H). Notably, the high scores of BDL and CCl_4_ mice were reduced significantly following treatment with PSS.

### 3.3 PSS up‐regulates MMP‐2 and down‐regulates TIMP‐1 in hepatic fibrosis

**Figure 3:**
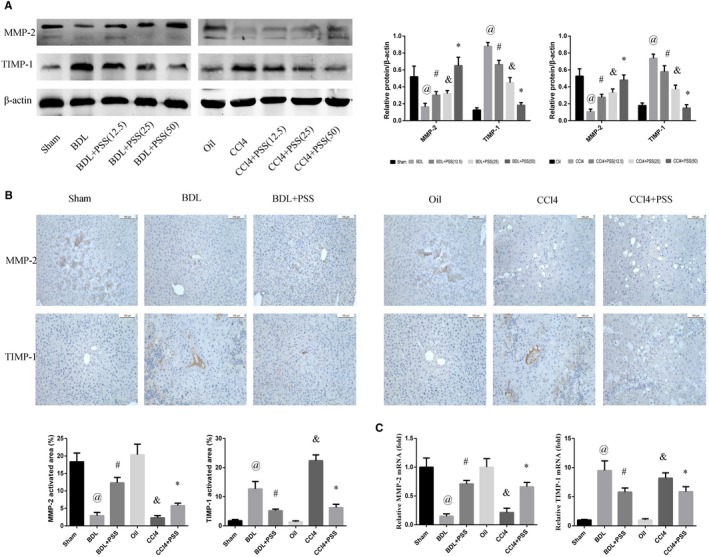
Propylene glycol alginate sodium sulphate up‐regulates MMP‐2 and down‐regulates TIMP‐1 in hepatic fibrosis. A, Western blots of MMP‐2 and TIMP‐1. Data are presented as means ± SD (n = 8). In BDL experiment, ^@^ *P* < .05 for BDL vs sham, ^#^ *P* < .05 for BDL + PSS (12.5) vs BDL. ^&^ *P* < .05 for BDL + PSS (25) vs BDL, *P < .05 for BDL + PSS (50) vs BDL. In CCl4 experiment, ^@^ *P* < .05 for CCl4 vs oil, ^#^ *P* (except the serum level of AST in CCl4 experiment) < .05 for CCl4 + PSS (12.5) vs CCl4. ^&^ *P* < .05 for CCl4 + PSS (25) vs CCl4, *P < .05 for CCl4 + PSS (50) vs CCl4. B, IHC staining of MMP‐2 and TIMP‐1 in liver tissues. C, The mRNA expression of MMP‐2 and TIMP‐1. Data of (B) and (C) were presented as means ± SD (n = 8). ^@^ *P* < .05 for BDL vs sham group, ^#^ *P* < .05 for BDL + PSS vs BDL group, ^&^ *P* < .05 for CCl4 vs oil group, *P < .05 for CCl4 + PSS vs CCl4 group

Next, the expression patterns of MMP‐2 and TIMP‐1 were examined in our fibrosis models. MMP‐2 protein expression was significantly decreased after the BDL operation or CCl_4_ injection, which was enhanced upon PSS treatment. Conversely, high TIMP‐1 levels observed in the BDL and CCl_4_ models were down‐regulated upon PSS injection, indicating alleviation of fibrosis (Figure 3A). Moreover, immunostaining with anti‐MMP‐2 and TIMP‐1 antibodies confirmed the expression patterns of MMP‐2 and TIMP‐1 in tissue samples from PSS‐treated mice (Figure 3B). MMP‐2 and TIMP‐1 mRNA expression were consistent with those of the corresponding proteins (Figure 3C).

### 3.4 PSS suppresses the TGF‐β1/Smad pathway in hepatic fibrosis

**Figure 4:**
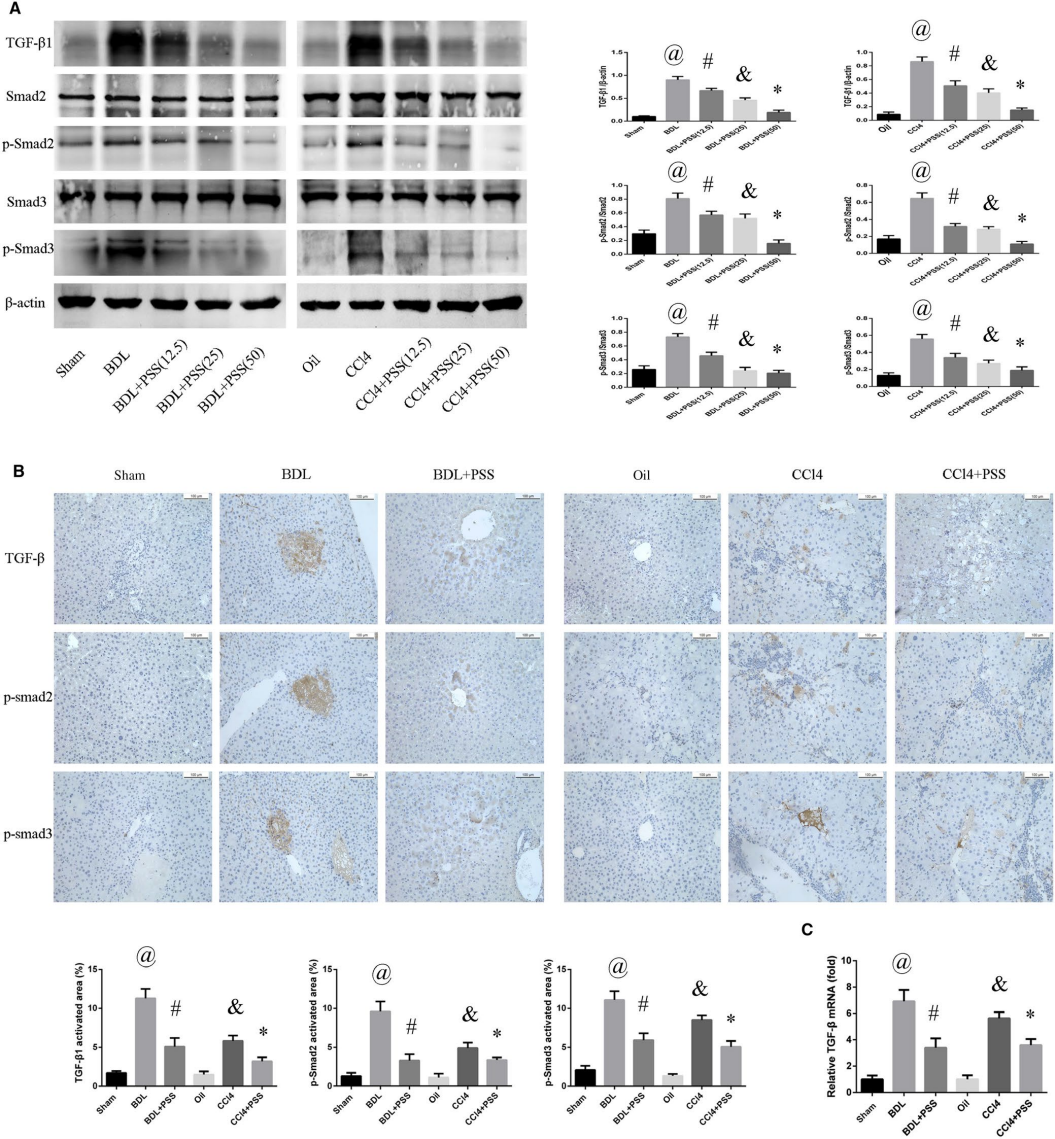
Propylene glycol alginate sodium sulphate suppresses hepatic fibrosis through the TGF‐β1/Smad pathway. A, Western blot analysis of TGF‐β1, Smad2, Smad3, p‐Smad2 and p‐Smad3 expression. Data were presented as means ± SD (n = 8). In BDL experiment, ^@^ *P* < .05 for BDL vs sham group, ^#^ *P* < .05 for BDL + PSS (12.5) vs BDL group. ^&^ *P* < .05 for BDL + PSS (25) vs BDL group, *P < .05 for BDL + PSS (50) vs BDL group. In CCl4 experiment, ^@^ *P* < .05 for CCl4 vs oil group, ^#^ *P* < .05 for CCl4 + PSS (12.5) vs CCl4 group. ^&^ *P* < .05 for CCl4 + PSS (25) vs CCl4 group, *P < .05 for CCl4 + PSS (50) vs CCl4 group. B, IHC staining of anti‐p‐Smad3, p‐Smad2 and TGF‐β1 antibodies. C, Expression of TGF‐β1 mRNA. Data of (B) and (C) were presented as means ± SD (n = 8), ^@^ *P* < .05 for BDL vs sham group, ^#^ *P* < .05 for BDL + PSS vs BDL group, ^&^ *P* < .05 for CCl4 vs oil group, *P < .05 for CCl4 + PSS vs CCl4 group

Phosphorylated Smad2 (p‐Smad2) and phosphorylated Smad3 (p‐Smad3) generated by TGF‐β1 lead to the activation of hepatic fibrosis.^1^ Accordingly, we evaluated p‐Smad2, p‐Smad3 and TGF‐β1 expression to determine the mechanisms underlying the anti‐fibrotic effects of PSS. In the BDL and CCl_4_ model groups, TGF‐β1, p‐Smad2 and p‐Smad3 levels were remarkably increased, which were suppressed following PSS treatment (Figure 4A). We further examined these factors in liver tissues to confirm Western blot findings (Figure 4B). As expected, PSS effectively reduced the expression levels of TGF‐β1, p‐Smad2 and p‐Smad3 (Figure 4B). Notably, the p‐Smad2 and p‐Smad3 in the liver tissues of BDL and CCl_4_ group accumulated in the nuclei when compared with the sham or oil group. But this tendency could be mitigated with PSS treatment. Consistently, mRNA expression of TGF‐β1 was decreased upon treatment with PSS after BDL operation or repeated CCl_4_ injection (Figure 4C).

### 3.5 PSS inhibits the JAK2/STAT3 pathway to exert anti‐autophagic effects in hepatic fibrosis

**Figure 5:**
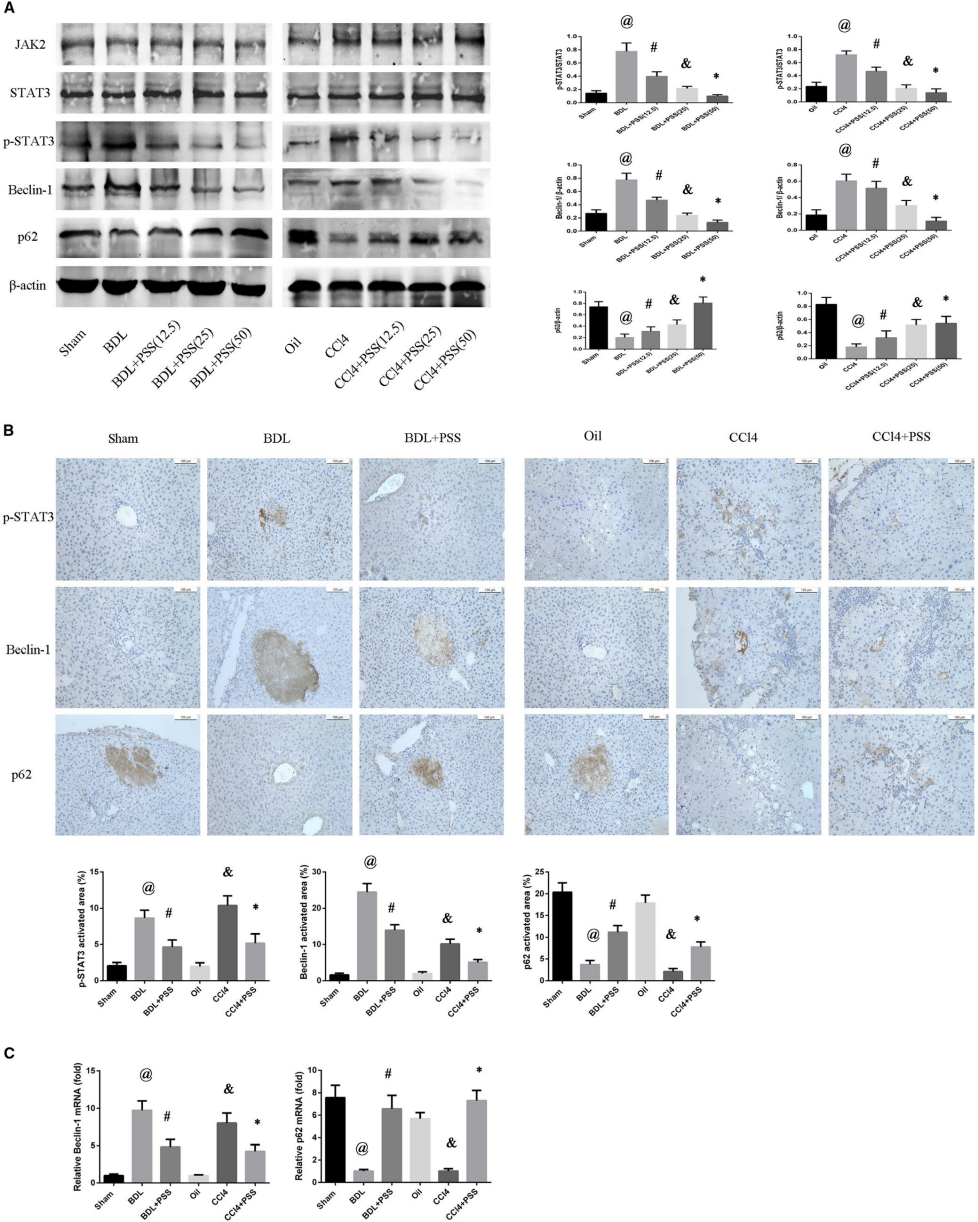
Propylene glycol alginate sodium sulphate inhibits the JAK2/STAT3 pathway to exert anti‐autophagic effects in hepatic fibrosis. A, Western blot analysis of JAK2, STAT3, p‐STAT3, Beclin‐1 and p62 expression. Data are presented as means ± SD (n = 8). In BDL experiment, ^@^ *P* < .05 for BDL vs sham group, ^#^ *P* < .05 for BDL + PSS (12.5) vs BDL group. ^&^ *P* < .05 for BDL + PSS (25) vs BDL group, *P < .05 for BDL + PSS (50) vs BDL group. In CCl4 experiment, ^@^ *P* < .05 for CCl4 vs oil group, ^#^ *P* < .05 for CCl4 + PSS (12.5) vs CCl4 group. ^&^ *P* < .05 for CCl4 + PSS (25) vs CCl4 group, *P < .05 for CCl4 + PSS (50) vs CCl4 group. B, IHC staining of anti‐p62, Beclin‐1 and p‐STAT3 in liver tissues. C, Expression of Beclin‐1 and p62 mRNA. Data of (B) and (C) were presented as means ± SD (n = 8), ^@^ *P* < .05 for BDL vs sham group, ^#^ *P* < .05 for BDL + PSS vs BDL group, ^&^ *P* < .05 for CCl4 vs oil group, *P < .05 for CCl4 + PSS vs CCl4 group

In view of the finding that autophagic death plays a significant role in progression of hepatic fibrosis, we examined the potential impact of the JAK2/STAT3 pathway on regulation of cellular autophagy. Suppression of JAK2/STAT3 pathway has been showed to ameliorate liver fibrosis in rat in a recent study.^28^ JAK2 is a novel regulator of TGF‐β, and TGF‐β/JAK2/STAT3 signalling is regarded as a non‐canonical TGF‐β signalling in fibrosis.^28^, ^29^, ^30^ Protein expression of JAK2 and total STAT3 was not significantly different among the groups, while BDL‐operated and CCl_4_‐treated mice showed enhanced expression of phosphorylated STAT3 (Figure 5A). STAT3 has been shown to be a major regulator of autophagy in some recent studies.^31^, ^32^ Beclin‐1, a key regulator of autophagy, tended to show a significant increase in both BDL and CCl_4_ models, which was markedly suppressed by PSS. Conversely, p62, inhibited in BDL‐operated and CCl_4_‐treated groups, was promoted upon synchronous PSS injection. Significant immunohistochemical staining of p‐STAT3 in liver tissues was observed in hepatic fibrosis model mice, and in particular, the expression of p‐STAT3 was highlighted in the nuclei. Meanwhile, PSS administration had a marked suppression effect (Figure 5B). The results of Beclin‐1 and p62 staining and RT‐PCR were consistent with Western blot data (Figure 5C).

### 3.6 PSS suppressed the activation of HSCs down‐regulated the activation of TGF‐β/Smad and JAK/STAT pathways in vitro

**Figure 6:**
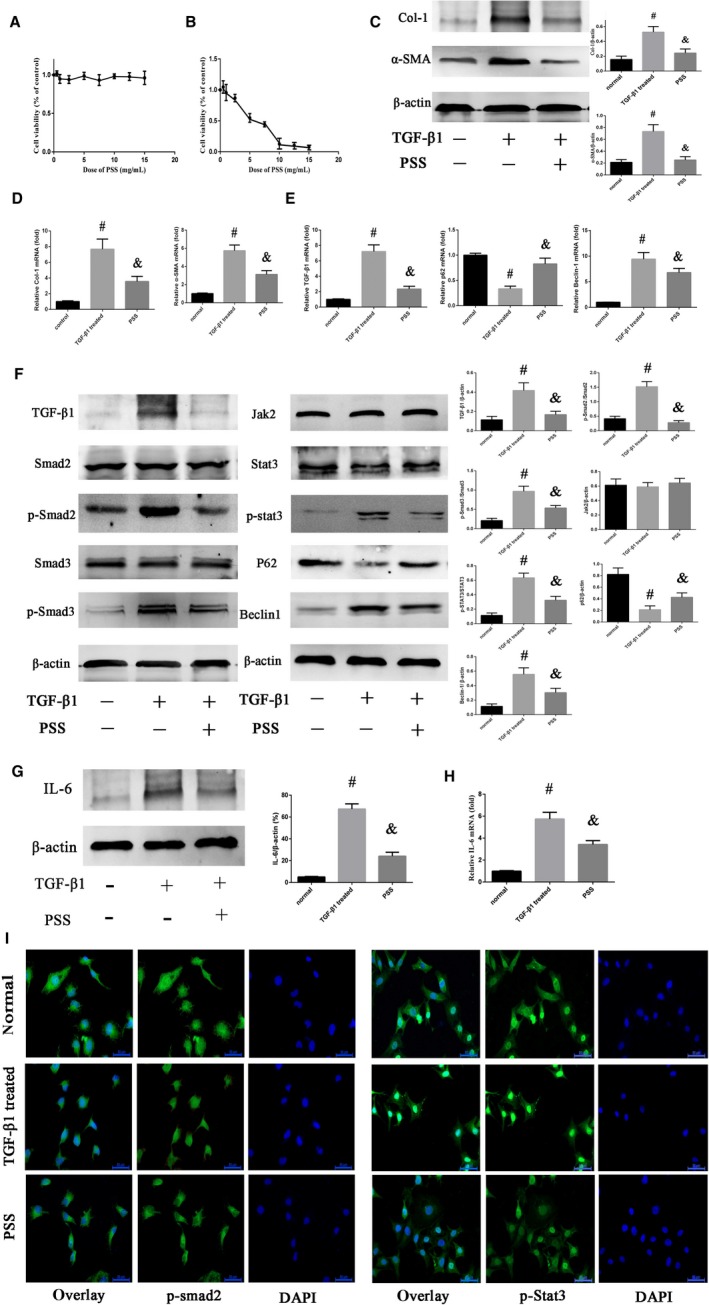
Inhibitory effects of PSS on HSC activation and TGF‐β/Smad and JAK/STAT pathways in vitro. A, The cytotoxicity examination of PSS in normal HSC cells. B, Cell viability after PSS administration in activated HSCs. C, The results of Western blot of Col‐1 and α‐SMA in normal, TGF‐β1–treated and PSS groups. D, mRNA expression of Col‐1 and α‐SMA. E, mRNA expression of TGF‐β1, P62 and Beclin‐1. F, Protein expression of TGF‐β1, Smad2, p‐Smad2, Smad3, p‐Smad3, Jak2, STAT3, p‐STAT3, P62 and Beclin‐1 was detected by Western blotting. G, Protein expression of IL‐6. H, mRNA expression of IL‐6. I, IF staining of p‐Smad2 and p‐STAT3 in LX‐2 cells (original magnification, 400×). Above experiments were all repeated three times. Data are presented as means ± SD. ^#^ *P* < .05 for TGF‐β1–treated group vs normal group; ^&^ *P* < .05 for PSS group vs TGF‐β1–treated group

To further reveal the inhibitory effect of PSS on hepatic fibrosis, we then used LX‐2 cells to perform in vitro studies. As shown in Figure 6A, PSS showed no significant cytotoxicity in normal HSCs. However, the viability of TGF‐β1–activated LX‐2 cells was significantly suppressed with PSS administration in a dose‐dependent way in this study, and the IC50 of PSS was 5.8 mg/mL according to the data (Figure 6B). Therefore, PSS of 5.8 mg/mL was used in subsequent experiments. As expected, enhanced expression of protein Col‐1 and α‐SMA after TGF‐β1 activation was reduced in the presence of PSS (Figure 6C) in LX2 cells. Then, we examined the mRNA expression of Col‐1 and α‐SMA, and the study showed that the mounting expression of Col‐1 and α‐SMA mRNA after activated by TGF‐β1 was down‐regulated in the PSS group (Figure 6D). We therefore confirmed PSS also possessed anti‐fibrosis function by suppressing the activation of HSCs in vitro.

Then, our study managed to investigate the effect of PSS on TGF‐β/Smad and JAK/STAT pathways in vitro. In Figure 6E, the mRNA expression of TGF‐β1 and Beclin‐1 was up‐regulated after the HSCs were activated, and PSS administration could down‐regulate TGF‐β1 and Beclin‐1 mRNA significantly. On the contrary, the decreasing p62 mRNA expression was prevented by PSS. The result of Western blot assay showed that the expression of TGF‐β1, p‐Smad2, p‐Smad3 and p‐STAT3 was up‐regulated in TGF‐β–treated group (Figure 6F), indicating TGF‐β/Smad and JAK/STAT pathways were activated after TGF‐β1 treatment in LX2 cells. In addition to TGF‐β1, IL‐6, a upstream of JAK2, can also directly stimulate the JAK2/STAT3 signalling.^33^, ^34^ In the result of Western blots, as the direct upstream factor of JAK2/STAT3, IL‐6 was up‐regulated in activated HSCs and the increasing level of IL‐6 was prevented by PSS significantly (Figure 6G). The examination of mRNA expression showed consistent results (Figure 6H). Two downstream proteins of p62 and Beclin‐1, known as the autophagic regulators, were also detected in this study. Consistently, p62 was restrained and Beclin‐1 promoted after the activation of HSCs. But these effects could be obviously suppressed in the presence of PSS. Besides, the IF results of p‐Smad2 and p‐Stat3 were consistent with the above experiments (Figure 6I). p‐smad2 accumulated in nucleus from cytoplasm in TGF‐β1–treated group when compared with the normal group. However, this effect was significantly mitigated in the PSS group. Similar results could also be detected in the IF staining of p‐Stat3. To sum up, PSS could suppress the activation of HSCs through down‐regulating the activation of TGF‐β/Smad and JAK2/STAT3 pathways to show its anti‐fibrosis function in vitro in our study.

## 4 DISCUSSION

**Figure 7:**
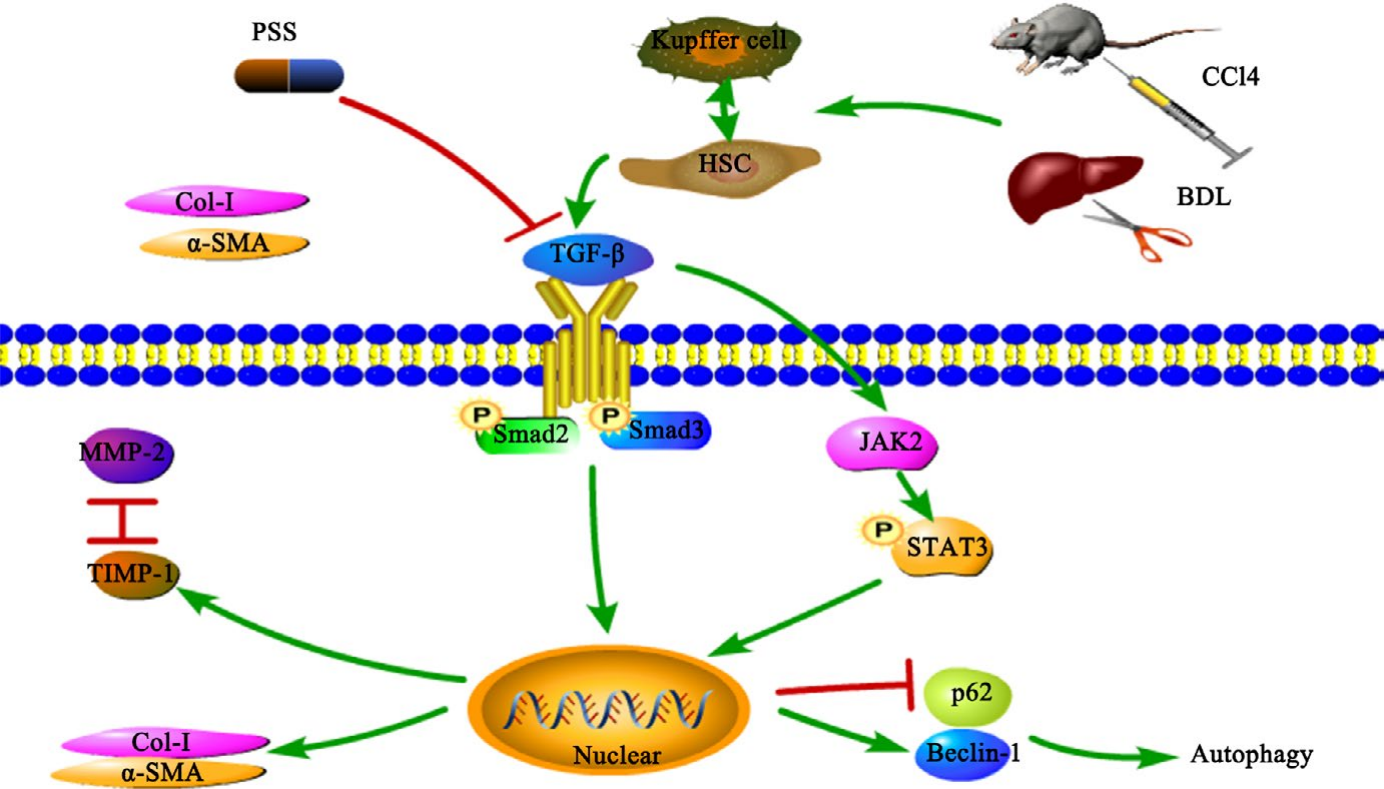
Potential mechanism of action of PSS against hepatic fibrosis in our models. After BDL operation or CCl4 injection, Kupffer cells are activated to secrete IL‐6 and HSCs were activated to produce TGF‐β. Through the TGF‐β1/Smad2/3 pathway, higher levels of Col I and α‐SMA are produced that induce excessive ECM accumulation. Higher Beclin‐1 and lower p62 levels are secreted through the JAK2/STAT3 pathway to induce cellular autophagy. Up‐regulation of TIMP‐1 is associated with hepatic fibrosis, and the TIMP‐1/MMP‐2 balance is altered to regulate production and elimination of the extracellular matrix. PSS significantly suppresses the production of TGF‐β1 and exerts anti‐fibrosis effects

In this study, we demonstrated the anti‐fibrotic activity of PSS and examined the mechanisms underlying PSS‐mediated hepatic fibrosis amelioration in our models (Figure 7). PSS suppressed inflammation and synthesis of ECM and inhibited activation of HSCs through a mechanism involving the TGF‐β1/Smad2/3 pathway. Furthermore, JAK2/STAT3 pathway–related autophagy was also identified as a potential therapeutic target for hepatic fibrosis in our study.

Chronic liver injury leads to hepatic fibrosis, which usually progresses to cirrhosis, hepatocellular carcinoma and liver failure.^22^ While significant progress has been made in clarifying the underlying mechanisms of hepatic fibrosis, no effective drugs or treatment methods are available at present,^36^ highlighting the urgent medical requirement for novel anti‐fibrosis strategies. PSS, an oral heparinoid with good anti‐coagulative, hypotensive and anti‐inflammation activities, has been used to treat patients with cerebrovascular, cardiovascular and other diseases in China for the past 30 years.^10^ The low cost and high efficacy of this compound make it an ideal choice as a long‐term therapeutic drug.^37^

Here, we used the widely accepted BDL and CCl_4_ mouse models and LX‐2 cell models to verify the protective effects of PSS. Inflammation is a critical regulator in hepatic fibrosis.^6^ Hepatic injury triggers inflammatory activity and releases cytokines to synthesize ECM through activating HSCs.^38^ And high level of TGF‐β1 and IL‐6 produced by Kupffer cells has been investigated to be important pro‐inflammatory factors in HSC activation.^6^ Activated HSCs, in turn, regulate inflammation and generate ECM and collagen.^38^ In our in vivo study, significant histological changes and high IL‐6 expression were reduced in the presence of PSS, which was further confirmed by Col‐1 and collagen fibres detections with Sirius red and Masson's trichrome staining. Increasing α‐SMA expression is also a crucial character of HSC activation.^6^ However, this expression pattern could be inhibited upon administration of PSS. Together with our in vitro study, these results supported the theory that PSS suppresses inflammation and HSC activation and promotes degradation of Col‐1 to inhibit hepatic fibrosis development.

TGF‐β1 secreted from HSC and Kupffer cells is the principal isoform of the TGF‐β family and a key profibrogenic cytokine in the development of hepatic fibrosis.^3^, ^38^, ^39^ Numerous signalling pathways are regulated by TGF‐β, and drugs that can intervene those pathways may have the potential anti‐fibrosis pharmacological effects.^41^ TGF‐β1 binds with TGF‐β type Ⅱ receptor, then activates the TGF‐β type Ⅰ receptor and eventually phosphorylates Smad2 and Smad3.^41^, ^42^ After phosphorylation, Smad2 and Smad3 can bind with Smad4 to form oligomeric complexes, which accumulate in the nucleus and affect the transcription of specific genes and their downstream factors.^43^, ^44^ In our study, we observed a significant increase in TGF‐β1, p‐Smad3, and p‐Smad2 levels in fibrotic mice, which were suppressed upon PSS administration. Recent studies advocate that TGF‐β1 is required to alter the balance between MMPs and TIMPs to affect matrix degradation.^38^ TGF‐β1 and its latency‐associated peptide (LAP) can bind to the latency TGF‐β1–binding protein‐1 (LTBP‐1) to form part of ECM.^46^ Moreover, latent TGF‐β1 is closely associated with MMP‐2 and MMPs are fibrolysis of proteolytic enzymes that can counterbalance fibrogenesis.^17^, ^46^ In contrast to MMP2, TIMP‐1 produced by TGF‐β1 can inhibit ECM degradation through the activation of Smad3.^48^ In our animal model, MMP‐2 protein and mRNA levels were suppressed, while those of TIMP‐1 were enhanced in fibrotic mice. Notably, altered expression patterns of these marker molecules were significantly abrogated upon PSS injection. Our results collectively indicate that the TGF‐β1 signalling pathway is involved in the anti‐fibrotic activity of PSS.

Accumulating evidence has established that autophagy is a major participant in numerous liver diseases, such as autoimmune hepatitis, hepatic ischaemia reperfusion injury and hepatocellular carcinoma.^49^ Although the autophagy of HSCs may moderate fibrogenesis in some studies, it saves energy for activated HSCs.^47^ Increased autophagic flux during HSC activation was recently shown to be involved in the pathogenesis of hepatic fibrosis.^15^, ^49^ Beclin‐1, together with autophagy‐related genes (ATGs) and class III phosphatidylinositol 3‐kinase (VPS34), forms a complex, which is necessary to the formation of autophagosome.^51^ As one of the autophagy adaptors, p62 sequesters the autophagosome by selectively targeting cargo to the autophagosome membrane.^51^ In our study, Beclin‐1 displayed significantly higher expression in the fibrotic model groups and p62 was simultaneously inhibited. Interestingly, PSS administration led to suppression of autophagy conditions, clearly indicative of anti‐autophagic activity.

We further demonstrated an important role of JAK2/STAT3 in fibrotic autophagy in our study. Both TGF‐β1 and IL‐6 have been shown to be regulators of JAK2/STAT3 pathway.^30^, ^34^ STAT3 is a potential transcription factor, and phosphorylated STAT3 may dimerize, translocate to the nucleus and mediate the transcription for extracellular signals after binding to DNA target sites.^52^ The pro‐autophagic effect of nuclear p‐STAT3 is associated with the modulation of hypoxia‐inducible factor 1, α subunit (HIF1A) in hypoxia and BCL2/adenovirus E1B 19 kD interacting protein 3 (BNIP3).^52^ HIF1A promotes the expression of BNIP3 and its ligands, which can strength the inductions of autophagosome.^52^ While the expression of total STAT3 and JAK2 proteins among these groups was not significantly different in our study, the p‐STAT3 level was promoted in fibrotic mice and activated LX‐2 cells, which was suppressed by PSS, as expected. These findings clearly demonstrate that PSS administration markedly inhibits the activation of the JAK2/STAT3 pathway, in turn, preventing autophagy, which may at least partially explain the mechanisms by which PSS exerts anti‐fibrotic effects and inhibits HSC activation.

Our results collectively suggest that PSS prevents hepatic fibrosis by suppressing inflammation, promoting ECM decomposition and inactivating HSCs through the TGF‐β1/Smad2/3 and JAK2/STAT3 pathways. Because of its multiple therapeutic effects, large number of sources and low cost, the natural extract PSS may effectively serve as a clinical supplement to treat hepatic fibrosis.

## 5 CONCLUSIONS

Propylene glycol alginate sodium sulphate exerts anti‐fibrotic activity in BDL and CCl_4_ mouse models, and in vitro. TGF‐β1/Smad2/3 and JAK2/STAT3 pathways are closely associated with the therapeutic effects of PSS, supporting its potential as an effective treatment agent for hepatic fibrosis.

## CONFLICT OF INTEREST

The authors declare no conflict of interest.

## AUTHOR CONTRIBUTIONS

S. Xu and Y. Mao designed the research; S. Xu, Y. Mao, J. Feng, J. Li and L. Wu performed the data analysis; S. Xu, Q. Yu, Y. Zhou, J. Zhang and J. Chen perform the animal experiments; Y. Mao, J. Ji, K. Chen, F. Wang and W. Dai conducted some other experiments; S. Xu wrote the manuscript; X. Fan and C. Guo edited the manuscript.

## Data Availability

Readers with reasonable requests can access the data supporting the conclusions of the study from the corresponding author or other authors.
